# Paced breathing and respiratory movement responses evoked by bidirectional constant current stimulation in anesthetized rabbits

**DOI:** 10.3389/fbioe.2022.1109892

**Published:** 2023-01-12

**Authors:** Xiaoyu Gu, Zixuan Guo, Maolin Cai, Yan Shi, Shoukun Wang, Fei Xie

**Affiliations:** ^1^ School of Biology and Medical Engineering, Beihang University, Beijing, China; ^2^ Medical School of Chinese PLA, Beijing, China; ^3^ School of Automation Science and Electrical Engineering, Beihang University, Beijing, China; ^4^ School of Automation, Beijing Institute of Technology, Beijing, China; ^5^ Department of Pulmonary and Critical Care Medicine, Chinese PLA General Hospital, Beijing, China

**Keywords:** airflow, airflow index, centroid frequency of diaphragm electromyography, diaphragm fatigue, diaphragm pacing

## Abstract

**Objective:** Diaphragm pacing (DP) is a long-term and effective respiratory assist therapy for patients with central alveolar hypoventilation and high cervical spinal cord injury. The existing DP system has some limitations, especially high price, inconvenience preoperative evaluation methods and diaphragm fatigue easily. Our objective was to develop a DP system and evaluated reliability through hardware testing and animal experiments.

**Methods:** A DP system with bidirectional constant current was designed, manufactured and tested. Effects of a wide range of stimulus amplitudes (range: .5–2.5 mA) and frequencies (range: 10–250 Hz) on airflow and corresponding inspired volume were investigated during DP. Differences in airflow characteristics under various stimulation parameters were evaluated using power function. ECG interference in diaphragm electromyography (EMGdi) was filtered out using stationary wavelet transform to obtain pure EMGdi (EMGdi_p_). 80-min period with a tendency for diaphragm fatigue by root mean square (RMS) and centroid frequency (*f*_*c*_) of EMGdi_p_ was studied.

**Results:** The increase of stimulus frequency and amplitude in animals resulted in different degrees of increase in envoked volume. Significant difference in Airflow Index (b) between anesthesia and DP provided a simple, non-invasive and feasible solution for phrenic nerve conduction function test. Increased stimulation duration with the developed DP system caused less diaphragm fatigue.

**Conclusion:** A modular, inexpensive and reliable DP was successfully developed. Its effectiveness was confirmed in animal experiments.

**Significance:** This study is useful for design of future implantable diaphragmatic pacemakers for improving diaphragm fatigue and convenient assessment of respiratory activity in experiments.

## 1 Introduction

DIAPHRAGM pacing (DP), as an effective respiratory assist therapy technique, has been used for half a century in patients with respiratory insufficiency caused by central hypoventilation syndrome (CHS) and high cervical spinal cord injury (Glenn and Phelps, 1985; Lo et al., 2013; Glenn et al., 1973). Each inspiration is formed by a train of electrical impulses that stimulate the phrenic nerve or diaphragm, causing the diaphragm to contract (Brouillette et al., 1983; Son et al., 2013). Compared to mechanical ventilation, DP improves patient speech, comfort, and mobility (DiMarco and Creasey, 2017). Currently, there are four main commercial diaphragm pacemakers throughout the world, of which three use radiofrequency transmission and one for percutaneous wire. However, these devices are expensive, costing US$50,000–60,000 for wireless transmission and US$20,000–25,000 for wired transmission (DiMarco and Creasey, 2017). Avery (United States), Synapse Biomedical (United States), Atrotech Oy (Finland) and Medimplant Inc. (Austria) are the main four manufacturers worldwide who have mastered the manufacturing technology of the device.

Whether DP can be successfully performed depends on preoperative and intraoperative assessment of phrenic nerve conduction and diaphragm function. Methods for corresponding functional evaluation are various, including diaphragm electromyographic (EMGdi) signal, phrenic nerve conduction velocity (7.5–9 ms) (Kenzi and Gandevia, 1985), transdiaphragmatic pressure (Pdi, Unilateral stimulation: 10 cm H_2_O) (Kenzi and Gandevia, 1985), and diaphragmatic movement under chest fluoroscopy (Diaphragm descent: > 5 cm) (Tedde et al., 2012). EMGdi and Pdi are based on compound muscle action potentials (CMAP), the acquisition of which requires dedicated and expensive physiological amplification instruments with extremely high sampling rate (10 kHz), complicated operation, and susceptibility to environmental interference (Sengupta-Ghosh et al., 2019; Luo et al., 1998). Pdi recording with two balloon catheters inserted into the stomach and esophagus are invasive and uncomfortable for the patient (Strongoli et al., 2010). In addition, repeated chest X-ray examinations will cause radiation damage to the body (Wall et al., 2006), which is not suitable for multiple follow-up of patients after DP.

In clinical and animal studies, artificial ventilation of DP has also been assessed by respiratory airflow (Geddes and Simmons, 1991; DiMarco et al., 2005; DiMarco et al., 2006). The airflow can be collected by the flow sensor connected to oral tube and mask, which is comfortable, cheap, convenient, and harmless (AL‐Khalidi et al., 2011). The tidal volume is calculated by integrating airflow to evaluate strength of respiratory muscle contraction and body metabolism. In addition, several studies have established a relationship between airflow and respiratory muscles (Arnal, 2018; Albani et al., 2021). The degree of inspiratory effort is identified by the concave and convexity of downward decay of inspiratory airflow curve, which is novel, non-invasive, and continuous (Albani et al., 2021). It is meaningful to extract the curve features of airflow for rehabilitation evaluation after DP.

Evaluating efficacy and safety of DP often involves the assessment of diaphragm fatigue (Onders, 2012), which is usually associated with stimulus waveform. In electrophysiological studies, the commonly used stimulation waveforms mainly include monophasic and biphasic pulse stimulation waveform. Some researchers have found that muscle fatigue is the result of polarization during electrical stimulation, which could be minimized by bidirectional current (Lilly et al., 1955). The bidirectional stimulation waveform is composed of anodic phase pulse and cathodic phase pulse. There is a time delay between the two phase pulses, so that the stimulation site does not affect the generation of action potential (Merrill and Jefferys, 2005). Animal experiments related to DP have also confirmed that bidirectional stimulation waveforms are less fatigued than unidirectional stimulation waveforms (Tanae et al., 1973), (Oda et al., 1981). Nevertheless, considering the low power consumption and reliability of the system, the stimulation waveform used in clinical practice with most commercial diaphragm pacemakers is unidirectional (Abraham et al., 2015; Headley et al., 2021).

The aim of this work was to develop an economical and reliable bidirectional constant-current stimulation DP system and to study its effects on respiratory activity through animal experiments. First, we developed the DP system using two microprocessors and defined the range of stimulation parameters. Output of stimulus waveforms was verified by hardware testing. Second, features of inspired airflow were extracted and the relationship with stimulation parameters was established. Finally, the effect of electrical stimulation on diaphragm fatigue was assessed by the changes in the time-frequency information of EMGdi.

## 2 Materials and methods

### 2.1 Hardware architecture and implementation of diaphragmatic pacemaker

**FIGURE 1:**
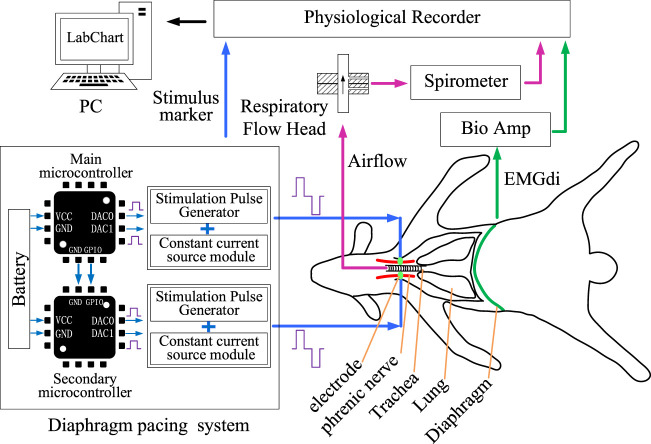
Design of DP system and acquisition of physiological parameters. DP system were developed by two microcontrollers. Effects of DP system on respiratory activity were verified by collecting airflow and EMGdi signals.

The application of DP techniques usually involves stimulation of the bilateral phrenic nerves or bilateral hemidiaphragm (Moxham and Shneerson, 1993; Alegret et al., 2019), which means that two independent stimulation channels need to be included in the DP system (Figure 1). Two independent, communicative, and identically circuited stimulators, named primary stimulator and secondary stimulator, constituted the control circuit of DP system. The hardware architecture of each stimulator mainly included power module, controller module, stimulation pulse generation module and constant current source module.

#### 2.1.1 Power supply

The stimulator was powered by 5 V through a lithium battery or an external step-down circuit. The 5 V voltage was then converted into −5, 3.3, and ±15 V according to the power supply requirements of different circuit modules. Since the working voltage of the microcontroller unit (STM32F091RxT, STMicroelectronics, Italy and France) was 3.3 V, low dropout linear voltage regulator (LT3042EDD#PBF, Analog Devices, United States) with high performance was used to convert 5–3.3 V. A DC-DC converter (E0515XT-1WR3, Mornsun, China) was applied to convert 5 V into 15 V and −15 V for power supply of the Op-Amp (ADA4625-2, Analog Devices, United States) in constant current source module. A charge pump converter (TPS60403QDBVRQ1, Texas Instruments, United States) converted 5 to −5 V to power the op amp (LTC1053CSW, Analog Devices, United States) in stimulation pulse generation module.

#### 2.1.2 Electrical stimulation controller

**FIGURE 2:**
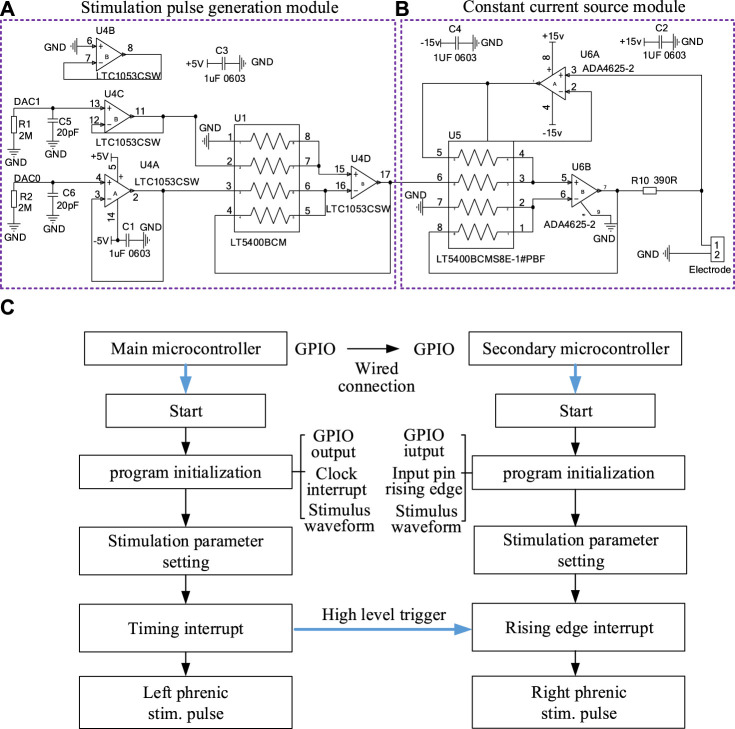
The hardware circuit of a stimulator in DP system and flow chart of the output of stimulation waveform. **(A)** Stimulation Pulse Generation Module and **(B)** Constant current source module of a stimulator. **(C)** The program execution flow of two microprocessors that output two stimulus waveforms synchronously.

For the microcontroller in each stimulator, two independent digital-to-analog converter (DAC) generated two same-direction voltage waveforms shown in Figures 1, 2. The stimulation pulse generation module amplified the voltage waveforms output by the DAC in two stages. The first-stage amplifying circuit amplified and inverted the two-channel voltage waveforms to generate positive-phase and negative-phase waveform. The second-stage operational amplifier circuit synthesized two opposite stimulation waveforms to obtain biphasic electrical stimulation waveforms.

#### 2.1.3 PCB and software design

A two-layer circuit board with less electrical noise and high reliability was used to design the stimulator. The upper board assembled the surface-mountable devices (SMDs) components of each module of the stimulator. In addition, OLED display in the upper circuit board displayed the stimulation parameters, including amplitude (0–10 mA, .01 mA resolution), pulse width (100–300 μs, 10 μs resolution), stimulation frequency (0–1,000 Hz, 5 Hz resolution), breathing cycle (0–50 times/min, 1 times/min resolution) and inspiratory-expiratory ratio (1:1–1:5, 1 resolution), which were regulated by five rotary coding switches (EC11, AISU, China) respectively. The circuit and PCB layout of our stimulator were designed by Altium Designer 20 (Altium, Ltd., San Diego, CA) and delivered to Jialichuang PCB for manufacturing.

The program for microprocessor was generated in the STM32CubeMX to configure specific functions for each pin, such as DAC and SPI communication. As shown in Figure 2C, the software was designed according to the execution process of the system. After program initialization and clock interruption, the microprocessor completed the DAC processed by the hardware constant current module to form stimulus waveform. In order to realize the synchronization of the two-way stimulation signals of the primary stimulator and secondary stimulator, GPIO ports of two microprocessors needed to be interconnected in hardware. The high level of the GPIO port of the primary microprocessor triggered the rising edge entry interrupt of the secondary microprocessor in software.

#### 2.1.4 Hardware test

The output terminal of two stimulators were connected to two identical resistors (15 kΩ). Voltage across test resistors were tested to verify the validity of stimulation parameter (1 mA amplitude, 160 μs pulse width, 25 Hz frequency, 30 times/min breathing cycle, 0.5 s inspiratory time) using an oscilloscope (MSOX4104A, KEYSIGHT, Malaysia).

### 2.2 Surgical procedure and data acquisition

Ten adult New Zealand white rabbits weighing 2.5–3 kg (male or female) were selected. The experimental animals were kept in the animal room of the Fourth Medical Center of the PLA General Hospital with free access to food and water (room temperature, 20°C ± 2°C, relative humidity, 50% ± 5%). All animal manipulations complied with the Helsinki International Declaration and were approved by the Biological and Medical Ethics Committee of Beihang University (Approval number, BM20210152).

#### 2.2.1 General anesthesia

After the animals were weighed, they were anesthetized with 3% sodium pentobarbital (1.2 mL/kg) through the ear vein. Anesthesia was considered successful when the pain response nearly disappeared. The animals were then fixed supine on the operating table. In experiment, the rabbit’s agitation was resolved by injecting an additional .5 mL of anesthetic through the ear vein.

#### 2.2.2 Isolation of phrenic nerve and recording of stimulation signals

As in the previous surgical procedure (Wen et al., 2018), the skin was cut along the medial border of the scapula. The brachial plexus was exposed after the muscle tissue was isolated. Phrenic nerve was found deep in the brachial plexus and dissociated with glass minute needles. Two Bipolar protective stimulation electrodes for transmitting stimulation signals were hung on the phrenic nerves on both sides and fixed with medical tape. The positive and negative output ends of an electrode wire were connected to the stimulator. Outputs of two stimulators were recorded by the physiological recorder (PL3516, PowerLab 16/35, ADInstruments, Sydney, Australia) to verify the effectiveness of stimulation signals sent to the phrenic nerve (Figure 1).

#### 2.2.3 Collection of airflow signals

A 4 cm incision was made in the midline skin of the rabbit’s neck. The trachea was bluntly dissected. An inverted “T” incision was made and an endotracheal tube was inserted. Tracheal tube was connected to the respiratory flow head (MLT10L, ADInstruments, Sydney, Australia) through a silicone hose with 6 mm inner diameter. A precision differential pressure sensor (FE141, Spirometer, ADInstruments, Sydney, Australia) was connected to flow head to collect respiratory airflow signals.

#### 2.2.4 EMGdi signals recording

The skin of the xiphoid process at the lower end of the animal’s sternum was incised, and a small incision of about 2 cm was cut along the linea alba to open the abdominal cavity.

Needle electrodes (MLA1213, ADInstruments, Sydney, Australia) inserted into the exposed diaphragm were connected to the Biological Amplifier (FE234, Quad Bio Amp, ADInstruments, Sydney, Australia) for collection of bilateral EMGdi signals (left EMGdi and right EMGdi).

#### 2.2.5 Phrenic nerve stimulation protocol

Two protocols were performed to assess effectiveness of DP and respiratory movement response in anesthetized rabbits.

Protocol 1 (*n* = 5). After the rabbits were deeply anesthetized, changes in inspired airflow and corresponding tidal volume were measured over a wide range of phrenic nerve stimulation amplitudes (.5–2.5 mA) and frequencies (10–250 Hz). The remaining respiratory parameters including pulse width (160 μs), inspiratory time (0.5 s) and respiratory rate (30 times/min) remained fixed throughout the study. Notably, the maximum current amplitude was limited to 2.5 mA to evaluate the effect of stimulation parameters on respiratory activity due to the limb movement around the brachial plexus caused by the excessive current. The train of stimuli delivered to the bilateral phrenic nerves determines the inspiratory time between .5 and 0.7 s to produce a full inspiration (Geddes and Simmons, 1991). For each set of stimulation parameters, airflow signals for four respiratory cycles were collected, of which four consecutive pacing cycles were used for analysis. Data generated by spontaneous breathing during DP were discarded.

Protocol 2 (*n* = 5), The ability of designed diaphragm pacemaker to maintain artificial ventilation was evaluated by repeated application of a fixed stimulation pattern for 80 min. Continuous intermittent electrical stimulation (.5 mA, 160 μs, 250 Hz, 0.5 s, 30 breaths/min) was delivered to bilateral phrenic nerves. The changes of bilateral EMGdi at different stimulation timings (0, 20, 40, 60, and 80 min) were monitored in each animal. EMGdi of four cycles of spontaneous breathing were taken for analysis after each stimulation timings. The RMS was used to evaluate the EMGdi amplitude (Phinyomark et al., 2009). Diaphragm fatigue analysis was performed based on the centroid frequency (*f*_*c*_) of power spectrum analysis (Rochester, 1985).

The physiological recorder recorded stimulation voltage values, airflow and EMGdi. Sampling rates of these signals were set to 40 kHz, 2000 Hz and 2000 Hz, respectively. Bilateral EMGdi were band-passed filtered between 0 Hz and 1000 Hz during acquisition. The processing of all signals was performed using the mathematical software Matlab (v. R2019a, Natick, MA, United States).

### 2.3 Airflow and EMGdi processing

#### 2.3.1 Airflow processing

##### 2.3.1.1 Calculation of breathing start and end time of airflow and tidal volume

For a segment of airflow, the zero-crossing criterion was used to extract the breathing start and end times of each breathing cycle (Schmidt et al., 1998). The portion of the airflow that exceeded the zero threshold was inhalation, and the opposite was exhalation. The airflow signals were integrated to obtain tidal volume for each breath (Geddes et al., 1990).

##### 2.3.1.2 Calculation of airflow index

The Airflow Index was calculated based on the waveform from the peak point in the inspiratory part of the airflow to the beginning of expiration. A non-linear equation represented by Eq. 1 was used to fit this curve. Differences in airflow changes under different stimuli were distinguished by Airflow Index.

$$\overset{\cdot }{V}=a\cdot t^{b}+c$$ (1)

Where, $\overset{\cdot }{V}$ represents inspiratory airflow, which is a time-dependent model. Parameters a and c are the rate of airflow reduction and airflow peak, respectively. The parameter b named Airflow Index is a dimensionless number describing the downward facing concavity of the inspiratory airflow curve. The upward and downward concavity of airflow curve reflect the activation of the inspiratory muscles (Albani et al., 2021). We calculated parameter b for each breath under various stimulus conditions. The parameter b less than 1 indicates that the respiratory airflow curve has a downward concavity, while parameter b greater than 1 has an upward concavity (Ranieri et al., 2000). The value of parameter b equal to 1 represents inspiratory flow decreases linearly.

#### 2.3.2 EMGdi processing

##### 2.3.2.1 Elimination of ECG interference in EMGdi

EMGdi signal acquisition was usually accompanied by ECG interference, and the interference amplitude on the left side was higher than that on the right side. P-wave and T-wave could be filtered out after 15 Hz high-pass filter. The remaining ECG in EMGdi was dominated by QRS complexes. Relevant studies have confirmed that the peak position of the QRS wave in the low-frequency coefficient of EMGdi after the stationary wavelet transformation is more obvious (Yang et al., 2013; Luo et al., 2018). Combination of QRS detection based on stationary wavelet transform and hard threshold filtering was applied to remove the ECG in EMGdi (Yang et al., 2013; Luo et al., 2018). The process of filtering ECG in EMGdi could be summarized in four steps. First, a four-scale stationary wavelet decomposition was conducted on the collected EMGdi. Second, we took square value of wavelet low-frequency coefficient of the fourth layer and then scaled it (scaling factor, k) to determine the range of ECG nosie. Third, the Donoho algorithm (Donoho, 1995) was adopted to determine the threshold and perform reverse hard threshold filtering to remove the ECG interference coefficient. After the coefficients were reconstructed, a pure EMGdi signal (EMGdi_p_) without ECG could be obtained. Of note, the method and process of cancelling ECG interference were same for left EMGdi and right EMGdi. The difference lies in the scaling factor (k). The value k of right EMGdi was higher than left EMGdi, and its value was 20–50.

##### 2.3.2.2 Feature extraction of EMGdi

The frequency and time domain features could be accurately assessed after removing ECG noise in left EMGdi (left EMGdi_p_) and right EMGdi (right EMGdi_p_). Changes in amplitude of left EMGdi_p_ and right EMGdi_p_ at each stimulation timings for each breath was evaluated by the RMS. The RMS was exprsessed as Eq. 2 using a moving window of 100 ms and overlap of 90%.

$$RMS=\sqrt{\frac{1}{n}\underset{n}{\sum }x_{n}^{2}}$$ (2)

Where *x*_*n*_ and n represent EMGdi and its corresponding length, respectively.

Muscle fatigue is related to changes in the frequency of the muscles, which tend to drop from high frequencies to low frequencies (Rochester, 1985). Power spectral density (PSD) was used to assess pure EMGdi (left EMGdi_p_ and right EMGdi_p_) for each breath by Welch method with a Hamming window (NFFT = 256). As a method of quantifying the power spectrum, the centroid frequency (*f*_*c*_) defined by Eq. 3 was used to assess diaphragm fatigue for each breath.

$$f_{c}=\frac{\sum f\cdot PSD\left(f\right)}{\sum PSD\left(f\right)}$$ (3)

Where *f* is frequency.

### 2.4 Data analysis and statistics

All relevant animal experimental results were expressed as mean ± standard deviation. Statistical differences (p) in Airflow index, RMS and *f*_*c*_ of EMGdi_p_ were analyzed using a paired *t*-test or Wilcoxon signed-rank test. Furthermore, the strength of the relationship between inspired tidal volume and Airflow index under different stimulation parameters was assessed by the Pearson correlation coefficient (r), and a regression analysis was conducted based on the least squares method. *p*-value less than .05 was considered a significant difference.

## 3 Results

### 3.1 Current stimulation output characteristics

**FIGURE 3:**
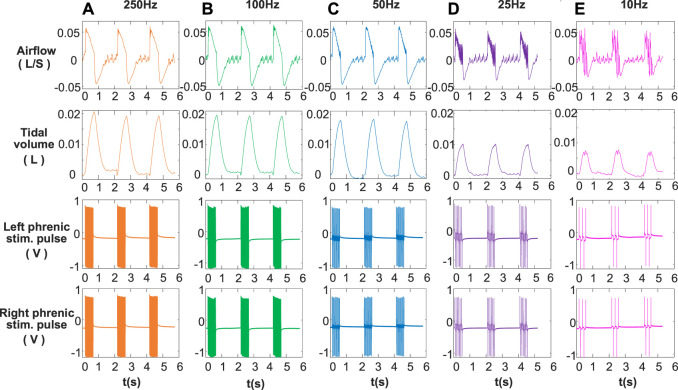
Recorded signals at different stimulation frequencies. **(A)** 250 Hz, **(B)** 100 Hz, **(C)** 50 Hz, **(D)** 25 Hz, **(E)** 10 Hz for airflow, tidal volume, left phrenic stim. pulse and right phrenic stim. pulse signals in a rabbit. Other respiratory stimulus parameters including amplitude, respiratory rate, pulse width, and inspiratory time were same, for 1 mA, 30 breath/min, 160 μs and 0.5 s respectively. The smoothness of the airflow waveform increases with stimulation frequency (10–250 Hz). Increasing the frequency of stimulation results in an increase in tidal volume.

The main purpose of designing DP system was to provide bidirectional constant-current stimulation pulses with a customized dual-channel synchronous output. Figure 3 shows the stimulus waveforms collected by physiological recorder at five different stimulus frequencies (10, 25, 50, 100, and 250 Hz) in animal experiments. Anodal stimulus pulses are followed by cathodal pulses of the same amplitude, which appear repeatedly to form a train of short-duration stimuli transmitted to the phrenic nerve. The stimulus pulse trains of the left phrenic nerve and the right phrenic nerve are confirmed to be synchronous. In Figure 3, the inspiratory time of artificial ventilation is same as .5 s, and different stimulus frequencies cause differences in the number of pulses. Number of pulses is 5 for 10 Hz stimulation frequency and 125 for 250 Hz. The voltage amplitudes of pulse trains with 1 mA recorded by the physiological recorder are basically same, indicating reliability of constant current stimulation of DP system.

Moreover, the voltage value of DP system for a stimulus parameter (1 mA, 160 μs, 30 breath/min, 25 Hz, .5 s) delivered to the 15 k resistive load was measured with an oscilloscope. The corresponding hardware test results are shown in the Supplementary Figures S1, S2. Similar to the test results in animal experiments, similarity of the two phase values, synchronization of two-channel stimulation waveforms, and precision of pulse width all show high reliability. In addition, the performance of DP system was also evaluated by acquiring diaphragmatic compound action potentials (CMAP) evoked by unidirectional constant current stimulation (Supplementary Figure S3). Both the developed system and the commercial device were effective in triggering CMAP and the waveform of it is consistent with previous studies (Luo et al., 1998).

### 3.2 Respiratory activity envoked by DP

To fully validate the operability of the developed DP system, phrenic nerve electrical stimulation tests in rabbits were performed. The effects of DP on inspired airflow and the corresponding tidal volume at 1 mA amplitude over a range of stimulation frequencies from 10 to 250 Hz are shown in Figure 3. It was observed that the inspiratory airflow curves were smooth when stimulation frequency was higher than 50 Hz. After inhalation elicited by the train of stimuli, the animal begins to exhale. From the airflow waveform, the transition between inspiration and expiration was good at each stimulation frequency. Below 50 Hz, single contraction of the diaphragm modulated respiratory airflow corresponding to left and right phrenic stim. pulses. At same stimulation amplitude (1 mA), tidal volume increased with the increase of stimulation frequency. The tidal volume generated by a full muscle contraction (50, 100, and 250 Hz) was much greater than that of a single muscle contraction (25 and 10 Hz).

**FIGURE 4:**
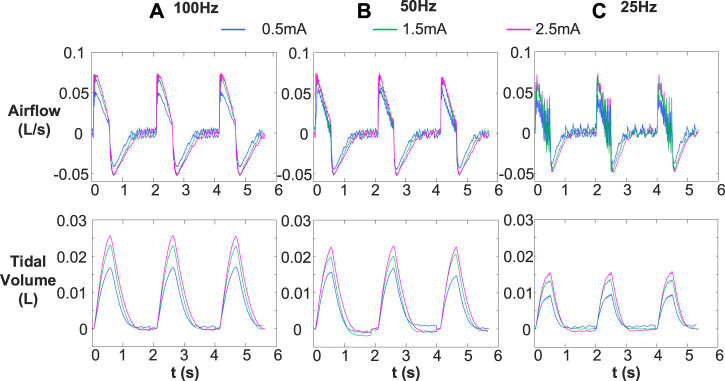
Comparison of inspired airflow and tidal volume under different stimulus frequencies [**(A)** 100 Hz, **(B)** 50 Hz, and **(C)** 25 Hz] with various stimulus amplitudes (.5, 1.5, an 2.5 mA). Other stimulus parameters were same, for 30 breath/min, 160 μs and .5 s, respectively. Different stimulation frequencies (25, 50, and 100 Hz) cause single and complete contraction of the diaphragm, and the corresponding tidal volume increase with the increase of stimulation frequency and amplitude.

Figure 4 represents the changes in airflow and corresponding tidal volume for various stimulation frequencies (25, 50, and 100 Hz) and stimulation amplitudes (.5, 1.5, and 2.5 mA). In the same conclusion as Figure 3, individual muscle contractions induced by stimulation frequency of 25 Hz (Figure 4C) did not change with the increase of stimulus intensity. The inspired tidal volume generated by both single and full contractions of diaphragm improved with increase of stimulus amplitude.

**FIGURE 5:**
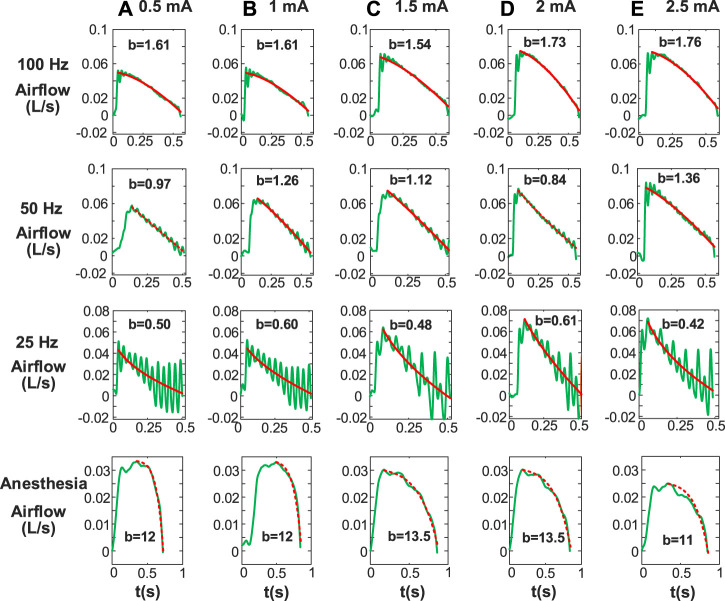
Airflow Index (b) for inspiratory phase including paced breathing and anesthesia. Stimulus amplitudes [**(A)** 0.5 mA, **(B)** 1 mA, **(C)** 1.5 mA, **(D)** 2 mA, **(E)** 2.5 mA] with various stimulus frequencies are different. Other stimulus parameters are same. b is obtained by fitting inspiratory airflow waveforms with Eq. 1. The b values under anesthesia are significantly different from that under diaphragm pacing.

Figure 5 shows changes in Airflow Index (b) for inspiratory phase during anesthesia and DP with wide range of stimulus frequencies and amplitudes. b values allow us to assess changes in characteristics of inspiratory airflow during DP with various parameters. It was observed that the power equation fit downward and upward concavity of the inspiratory airflow curve well under all conditions (DP and anesthesia). The airflow curve was not smooth due to individual contraction of the diaphragm at the stimulus frequency of 25 Hz, but the line fitted by the power equation could accurately reflect downward concavity of inspiratory airflow. For each stimulus frequency of DP, little differences between b values of inspiratory airflows caused by different stimulus amplitudes were found. These b-values are concentrated in a certain range. For example, DP (100 Hz) at stimulus amplitudes of .5, 1, 1.5, 2, and 2.5 mA envoked b values of inspiratory airflows for 1.61, 1.61, 1.54, 1.73, and 1.76, respectively. Moreover, at any given stimulus amplitude, the b values of inspired airflow during DP increased with stimulus frequency. b values ranged from .42 to .61 at stimulus frequency 25 Hz during DP, 50 Hz for .84–1.36, and 100 Hz for 1.54–1.76. The degree of activation of inspiratory muscles caused by different stimulation frequencies is different, which also explains the reason for the significant difference in the b value of the generated airflow. In addition, The b values of airflow under anesthesia (11–13.5) were much higher than those during DP (.42–1.76).

To further investigate Airflow Index difference between DP and anesthesia, we calculated the mean and standard deviation of b values of corresponding airflow. As shown in Table 1, at a stimulus frequency of 25 Hz, mean airflow index (b) corresponding to airflow envoked by wide range of stimulus amplitudes during DP was less than 1. At any stimulus amplitude, b values corresponding to airflow generated by DP at 50 Hz, 100 Hz and 250 Hz were all greater than 1. Furthermore, mean b value of airflow under anesthesia was 7.73 ± 3.22, which was much higher than mean b value under any stimulus parameter of DP shown in Table 1. Such significant differences provide a simple, non-invasive method of assessment of phrenic nerve conduction function testing.

**TABLE 1:** Mean Airflow Index (b) calculated for inspiratory airflow waveforms during DP and anesthesia status.

Frequency	25 Hz	50 Hz	100 Hz	250 Hz	Anesthesia
Amplitude (mA)					
.5	.81 ± .13	1.04 ± .15	1.68 ± .46	1.85 ± .41	7.73 ± 3.22
.75	.67 ± .17	1.06 ± .14	1.72 ± .34	1.82 ± .39	
1	.83 ± .14	1.34 ± .30	1.85 ± .39	1.86 ± .52	
1.25	.75 ± .11	1.06 ± .16	1.63 ± .32	1.78 ± .45	
1.5	.70 ± .14	1.29 ± .20	1.48 ± .63	1.86 ± .53	
1.75	.76 ± .08	1.16 ± .20	1.78 ± .53	1.61 ± .59	
2	.60 ± .13	1.56 ± .39	2 ± .27	1.81 ± .47	
2.25	.73 ± .21	1.28 ± .43	1.75 ± .53	1.80 ± .51	
2.5	.78 ± .19	1.31 ± .34	1.86 ± .27	1.70 ± .42	

Stimulus frequencies and stimulus amplitudes are varied.

**FIGURE 6:**
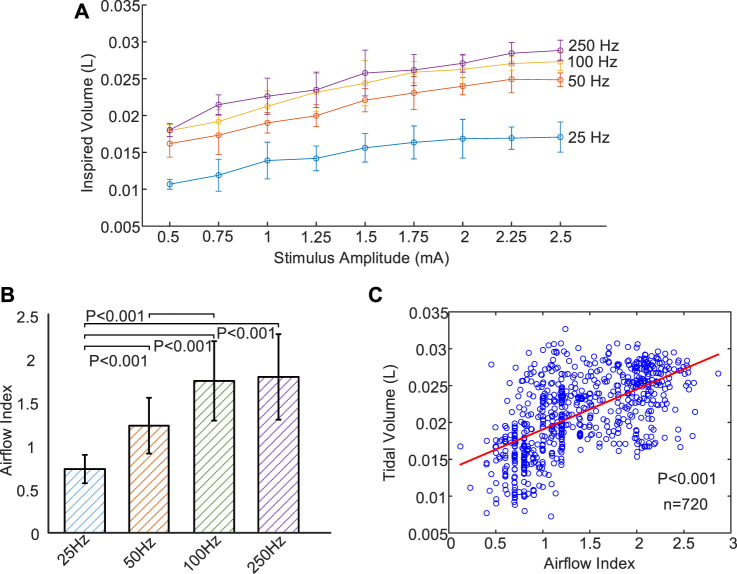
Changes of inspired volume and Airflow Index under different stimulus parameters. **(A)** Relationship between evoked tidal volume and incremental stimulus amplitudes for four stimulus frequencies (25, 50, 100, and 250 Hz). Inspired volume gradually increase with increase of stimulus current until it basically reached saturation at 2 mA. **(B)** Mean Airflow Index during DP for four different stimulus frequencies (25, 50, 100, and 250 Hz). B values increase with increasing stimulus frequency. **(C)** Correlation analysis between Airflow Index and inspired volume using scatter plot. The least squares regression line is represented by a solid red line. Strong correlation (*r* = .60, *n* = 720, *p* < .001) is observed between Airflow Index and inspired volume.

Figure 6A illustrates the mean and standard deviation of inspired tidal volume at various stimulus frequencies (25–250 Hz) and amplitudes (.5–2.5 mA). At each stimulus frequency during DP, the value of inspired volume gradually increased with increase of stimulus current until it basically reached saturation at 2 mA. In addition, at any stimulus current, inspired tidal volume during DP with 250 Hz were obviously higher than those generated at low frequencies (25, 50, and 100 Hz). Especially when the stimulus frequency was adjusted from 25 Hz to 50 Hz, the increase in tidal volume during DP was very pronounced at all stimulus currents (.5–2.5 mA). For example, DP (2 mA) at frequencies of 25, 50, 100, and 250 Hz aroused mean volume for .017 ± .003, .024 ± .001, .026 ± .002 and .027 ± .001 L, respectively.

Figure 6B is a comparison of mean Airflow Index (b) at various stimulus frequencies for all stimulus amplitudes during DP. These b values allow us to assess differences in airflow features at different stimulus frequencies. It can be observed that b values increased with increasing stimulus frequency. In this instance, mean b values of inspired airflow at frequencies of 25, 50, 100, and 250 Hz were .74 ± .16, 1.23 ± .32, 1.75 ± .46 and 1.80 ± .49, respectively. No Significant difference was observed between 100 and 250 Hz corresponding to b values (*p* = .21). Significant differences with *p* < .001 for the ramaining b values were found. Figures 6A, B display that both tidal volume and b values increases are associated with an increase in frequency. Exploring the relationship between b value and tidal volume can further verify significance of b value in evaluating effect of DP. Figure 6C shows the comparison of Airflow Index and inspired volume revealing strong correlation (*r* = .60, *n* = 720, *p* < .001) based on least squares regression analysis. The value of tidal volume reflects the degree of activation of the inspiratory muscles. The strong correlation between airflow index and tidal volume further confirms the reliability of airflow index in assessing the degree of activation of inspiratory muscles caused by DP.

### 3.3 Assessment of diaphragm fatigue induced by DP

**FIGURE 7:**
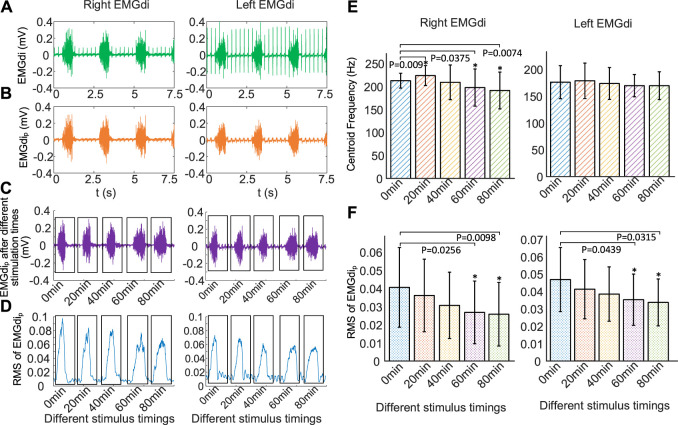
Changes of EMGdi after different stimulation time using the same stimulation parameters (0.5 mA, 250 Hz, 160 μs, 30 times/min, 1:3). **(A)** EMGdi signals, **(B)** EMGdi_p_ signals without ECG, **(C)** EMGdi_p_ after stimulation time of 0, 20, 40, 60, and 80 min, respectively, **(D)** RMS for the EMGdi_p_ shown in **(C)**, increasing the duration of DP results in a gradual decrease in EMGdi, **(E)** mean centriod frequency (*f*_*c*_) and **(F)** RMS of EMGdi_p_ after different stimulation time for five rabbits.*p < .05 in comparison to 0 min values. The increase of stimulus time makes the mean RMS of EMGdip (right EMGdi_p_ and left EMGdi_p_) decrease.

Figure 7A illustrates the time-domain information of right EMGdi and left EMGdi for three breathing cycles of a rabbitunder anesthesia. The amplitude of ECG interference in the left EMGdi was significantly higher than that in the right EMGdi. Pure EMGdi without ECG (EMGdi_p_) allows us to accurately extract its features. Figure 7B shows the left EMGdi and right EMGdi after filtering out ECG interference (left EMGdi_p_, right EMGdi_p_). It can be found that ECG interference in the left EMGdi and right EMGdi were effectively removed based on the stationary wavelet transform.

Figure 7C represents right EMGdi_p_ and left EMGdi_p_ for a single respiratory cycle at different stimulation times of 0, 20, 40, 60, and 80 min. Figure 7D is the result of using RMS to quantify right EMGdi_p_ and left EMGdi_p_ shown in Figure 7C. It was found that increasing the duration of DP resulted in a gradual decrease in EMGdi.

Figure 7E shows changes in *f*_*c*_ of mean right EMGdi_p_ and mean left EMGdi_p_ over different stimulus times during DP (0.5 mA, 250 Hz, 160 μs, 30 times/min, 1:3). Compared with no electrical stimulation, *f*_*c*_ of right EMGdi_p_ stimulated for 40, 60, and 80 min decreased by 1.73%, 7.07%, and 10.04%, respectively. Correspondingly, *f*_*c*_ of left EMGdi_p_ were reduced by 1.38%, 3.69% and 3.72%. However, compared to before electrical stimulation, *f*_*c*_ of right EMGdi_p_ and left EMGdi_p_ slightly increased by 5.15% (*p* = .009) and 1.44% (*p* = .52) respectively at stimulation times of 20 min. When comparing *f*_*c*_ of right EMGdi_p_ before stimulus, significant differences were observed at stimulation times of 60 min (*p* = .0375) and 80 min (*p* = .0074), but no significant difference were found at 40 min (*p* = .6075). When comparing *f*_*c*_ of left EMGdi_p_ before stimulation, no significant differences (*p* = .5160, *p* = .4812, *p* = .1125, *p* = .1084) were observed for any stimulus timings. The left diaphragm has a tendency to fatigue, but it is not significant, which means that the left diaphragm did not experience obvious fatigue during the 80-min stimulation time.

Figure 7F displays changes in the mean RMS of right EMGdi_p_ and left EMGdi_p_ after various DP times. It can be found that the increase of stimulus time made the mean RMS of EMGdi_p_ (right EMGdi_p_ and left EMGdi_p_) decrease. Compared with before stimulation, the mean RMS of right EMGdi_p_ with DP for 20, 40, 60, and 80 min decreased by 11.08% (*p* = .3369), 24.63% (*p* = .1404), 33.99% (*p* = .0439) and 36.45% (*p* = .0315). Correspondingly, the mean RMS of left EMGdi_p_ was reduced by 11.97% (*p* = .1368), 17.74% (*p* = .0531), 24.57% (*p* = .0256) and 27.99% (*p* = .0098). With the increase of stimulation time, the decrease of RMS of EMGdi means a significant decrease of diaphragm contractility.

## 4 Discussion

Compared with traditional ventilators, DP as another treatment for respiratory support, has the advantages of reduced respiratory infections, increased mobility, and improved living comfort (Whiteneck et al., 1992; DiMarco and Creasey, 2017; Headley et al., 2021). The high price of commercial diaphragm pacing systems limits its corresponding basic scientific research. Procurement process for parts is also cumbersome (DiMarco and Creasey, 2017). The integrity of preoperative and intraoperative phrenic nerve conduction is the key to successful DP. Measurement of phrenic nerve conduction velocity, changes in transphrenic pressure and diaphragmatic activity under fluoroscopy has the disadvantages of invasiveness and complicated operation (Kenzi and Gandevia, 1985; Tedde et al., 2012). In addition, the use of bidirectional electrical stimulation waveforms is an effective means to avoid muscle fatigue and prolong stimulation time (Sato et al., 1970).

In this study, we have developed a modular, inexpensive, and reliable dual-channel DP system that is easily implemented in other laboratories. Although the stimulus parameters of this system are customized to meet the physiological characteristics of breathing, it can be easily changed by modifying microprocessor program to meet specific animal experimental needs. As shown in Table 2, affordable and reliable electronic components were used to design DP system. The reliability of the output stimulus parameters of developed DP system was verified by recorded signals in circuit hardware tests and animal experiments (Figures 3, 4). The price to develop DP system is about $310, which is a very small expenditure for many scientific research institutions. Furthermore, the system is assembled based on SMDs components and is easy to manufacture, which is a good choice for many laboratories engaged in neurophysiological behavioral research. The current commercial DP systems used in clinics are usually wireless charging, which are mainly composed of external controllers, external transmitter and internal receivers (Talonen et al., 1983; DiMarco and Creasey, 2017). The external transmitter and internal receiver realize wireless power supply based on the principle of electromagnetic induction (Talonen et al., 1983). The system we developed provides an economical and feasible solution for future development of internal receivers for wireless transmission DP systems. Moreover, commercial electrophysiological stimulators with multiple channels are expensive. We provide a feasible and economical hardware design scheme for animal experiments that require multi channels to simultaneously deliver constant current stimulation.

**TABLE 2:** Development details of DP system and its working parameters.

Name of item	No. of item	Item price (USD)	Working parameters
STM32F091RxT Microcontroller	2	30.77	Current amplitude: 0–10 mA in .01 mA steps
Stimulation pulse generation module:			
LTC1053CSW OP-Amp	2	19.97	
LT5400BCM Resistor network	2	12.57	Pulse width: 100 µs−300 μs in 10 μs steps
Constant current source module:			
ADA4625-2 OP-Amp	2	8.58	
LT5400BCMS8E Resistor network	2	14.05	Stimulation frequency: 0–1,000 Hz in 5 Hz steps
Power module:			
TPS60403-Q1 Voltage regulator	2	15.53	
REF3433IDBVR Voltage reference	2	19.97	Breathing cycle: 0–50 times/min in 1 times/min steps
LT3042EDD Voltage regulator	2	7.54	
E0515XT-1WR3 Isolated module	2	5.18	
Display module	2	7.40	Inspiratory-expiratory ratio: 1:1–1:5 in 1 steps
Others (PCB, switches, passive components, etc.)	2	13.76	
	Total price		310.64

In order to have a complete validation of the feasibility of DP system, effects of various stimulation parameters on respiratory airflow were investigated. Meanwhile, we applied a bidirectional constant current electrical stimulation waveform to the phrenic nerve for its advantage of relieving diaphragm fatigue (Sato et al., 1970; Tanae et al., 1973). The stimulation parameters for DP include stimulus amplitude, pulse width, frequency, inspiratory time, and respiratory cycle. Inspiratory times ranging from .5 to 1.5 s are adequate for ventilation in most species (Geddes et al., 1990). After inspiratory airflow has ceased, prolonged stimulation time is not necessary (Geddes et al., 1990). In this work, we gave each rabbit a ventilation time of .5 s per breathing cycle. Changes in airflow and corresponding inspired tidal volumes at various stimulus frequencies and amplitudes during DP were studied. Consistent with previous findings (Peterson et al., 1986; Geddes et al., 1990), increases in stimulus frequency and amplitude caused an increase in inspired volume (Figures 3, 4), which further demonstrated the effectiveness of DP system we have developed. The airflow waveform produced by single contraction of the diaphragm is rough and jagged (Geddes et al., 1990). Our study showed that at a stimulation frequency of 25 Hz, the waveform of respiratory airflow showed that the diaphragm did not fully contract (Figures 3–5). This is because acute stimulation of the phrenic nerve preferentially activates large axons that innervate fast-twitch muscle fibers (DiMarco, 2009). As stimulation time increases, individual contractions will be replaced by smooth, coordinated contractions, even at low stimulation frequencies (Glenn et al., 1980; Glenn et al., 1984).

Considering the invasive injury and operational complexity of DP to verify phrenic nerve conduction function (Luo et al., 1998; Wall et al., 2006; Strongoli et al., 2010), Airflow Index (b) of inspired airflow was extracted. To the best of our knowledge, this is the first report on calculating changes in Airflow Index (b) of inspired airflow based on a power equation during DP. b values during anesthesia was much greater than that during DP (Figure 5; Table 1). b values generated by full contraction of diaphragm during DP (50, 100, and 250 Hz) were significantly higher than that of single contraction envoked by 25 Hz (Figure 6B). Compared with the traditional phrenic nerve function test, Airflow Index is a simple and intuitive indicator of efficient delivery of stimulating current to the diaphragm. Moreover, technical problems of commercial DP system, such as battery power failure and dysfunction of the electrode-nerve interface (DiMarco and Creasey, 2017), cause the reduction of inspired volume, which requires monitoring of inspired volume. The strong correlation between airflow index and inspired volume (Figure 6C) provides a possible solution for DP monitoring based on b values.

Another key factor in the long-term use of DP system is whether the diaphragm is fatigued (Oda et al., 1981). In the current research, diaphragm fatigue have been assessed by tidal volume changes (Tanae et al., 1973; Oda et al., 1981), which is not able to be assessed intuitively and accurately. Muscle fatigue can be evaluated by downward shift in *f*_*c*_ of EMG (Rochester, 1985). Filtering out ECG interference in EMGdi can more accurately quantify amplitude and frequency of EMGdi. The stationary wavelet transform was proven to be effective in removing ECG interference in EMGdi (Figure 7B). In this work, we used *f*_*c*_ of EMGdi_p_ to assess diaphragm fatigue within 80 min during DP, which is simple and reliable. With the increase of stimulation time, the amplitude and *f*_*c*_ of EMGdi showed a downward trend. Consistent with previous studies (Tanae et al., 1973), bidirectional constant-current electrical stimulation had a tendency to slightly fatigue the diaphragm in acute trials. The evaluation of diaphragm fatigue properties after DP by *f*_*c*_ of EMGdi_p_ is a further complement to previous studies. Simultaneously, the result further confirmed the effectiveness of our designed DP system.

## 5 Conclusion

In summary, we have proposed a modular, low-cost and reliable DP system with bidirectional constant current. In animal experiments with DP, significant difference in Airflow Index calculated by power function between anesthesia and DP provides a simple, non-invasive and feasible solution for phrenic nerve conduction function test. Moreover, DP was successful in pacing rabbits over 80-min period with a tendency for diaphragm fatigue by centroid frequency (*f*_*c*_) of EMGdi_p_. This study may be useful for the design of future fully implantable diaphragmatic pacemakers and convenient assessment of respiratory activity in animal and clinical experiments.

## 6 Limitations

There are some limitations in our study. First, to prevent wound infection, the stimulator needs to be fully implanted in the body. Implanting batteries in the body for power is not safe and requires battery replacement. We did not design a wireless charging solution for the DP system. The wireless power supply module should be introduced on the basis of the hardware design of this research in future. Second, we conducted acute experiments of 80 min of phrenic nerve stimulation to study the fatigue properties of the diaphragm. In future research, after developing a wirelessly charged DP system, chronic implantation animal experiments (7–15 days) are required. Further validation of respiratory characteristics by adding histological testing is necessary. Third, responses to respiratory activity elicited by different stimulus waveforms are different. The waveform used for DP in this study was bidirectional constant current waveform. Exploring effects of multiple different waveforms on respiratory activity should also be the focus of future research. Finally, we demonstrated the potential feasibility of a low-cost DP system. In human efficacy trials, a large number of clinical trials will increase the price of a commercial product. The price of the developed DP system needs to be further evaluated based on the cost of future efficacy trials on humans.

## Data Availability

The raw data supporting the conclusion of this article will be made available by the authors, without undue reservation.
