# Supplementary material for: Paced breathing and respiratory movement responses evoked by bidirectional constant current stimulation in anesthetized rabbits
Source: Front Bioeng Biotechnol. 2023 Jan 12;10:1109892. doi: 10.3389/fbioe.2022.1109892 (PMC9877234; doi:10.3389/fbioe.2022.1109892)
Supplement: Supplementary file 1 [file Presentation1.pdf]

SUPPLEMENTARY FILE

Additional file 1.

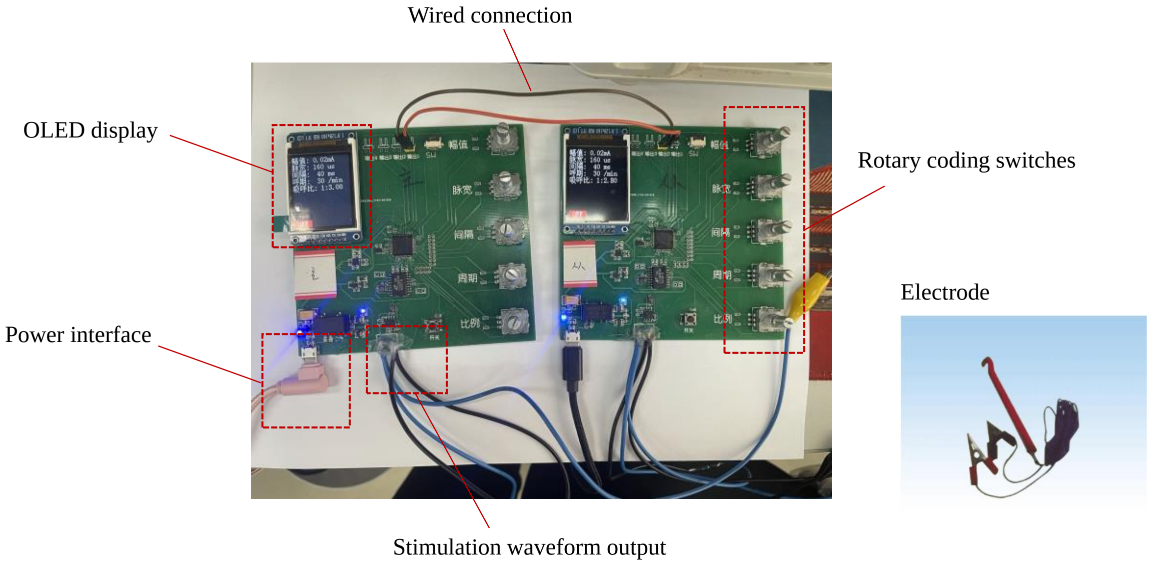

Figure S1 The proposed diaphragm pacing system.

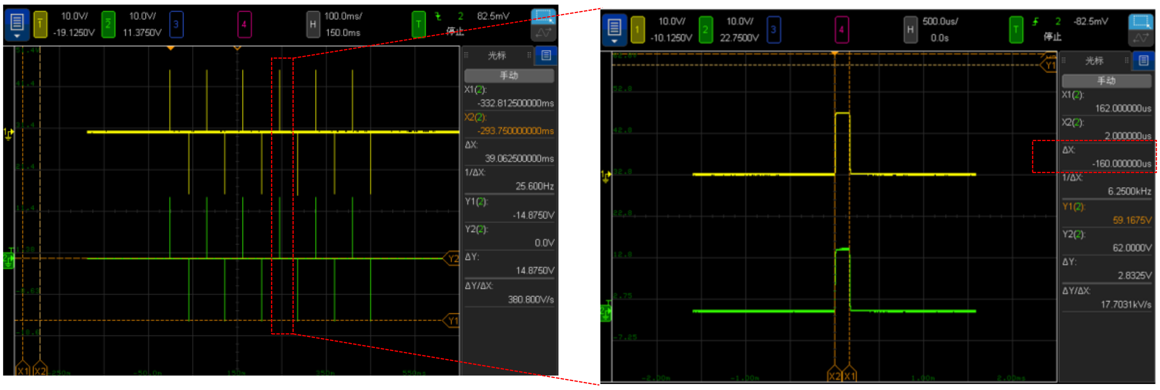

Figure S2 Hardware test for developed DP system. Stimulus parameters: 1mA, 160μs, 25Hz, 30/min, 0.5s

Additional file 2.

We modified the microprocessor program of one of the modules in the DP system, and changed the bidirectional waveform into unidirectional output, which was used to verify the reliability/performance of the developed system against commercial device(FE180, ADInstruments, Australia). Figure S3 shows the comparison of diaphragmatic compound action potentials (CMAP) evoked by unidirectional pulse stimulation with the developed system and the commercial device. The stimulation intensity, pulse width and frequency were 2.5mA, 200μs and 1Hz respectively. It is found that both the developed system and

the commercial device were effective in triggering CMAP. The amplitude of action potentials triggered by these two systems ranged from 2.50 mv to 3.20 mv.

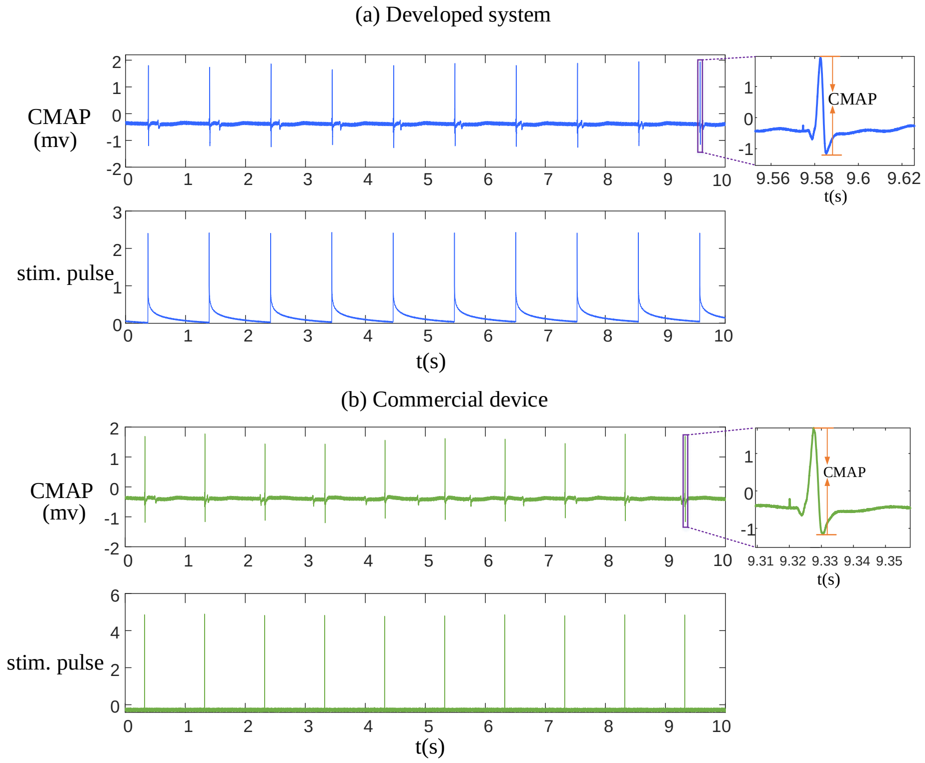

Figure S3 Comparison of diaphragmatic compound action potentials (CMAP) evoked by unidirectional pulse stimulation of the developed system and a commercial device.
